# Comparison of Compressed Sensing and Gradient and Spin-Echo in Breath-Hold 3D MR Cholangiopancreatography: Qualitative and Quantitative Analysis

**DOI:** 10.3390/diagnostics11040634

**Published:** 2021-04-01

**Authors:** Weon Jang, Ji Soo Song, Sang Heon Kim, Jae Do Yang

**Affiliations:** 1 Department of Radiology, Jeonbuk National University Medical School and Hospital, Jeonju 54907, Korea; weon0315@gmail.com (W.J.); buddyya@daum.net (S.H.K.); 2 Research Institute of Clinical Medicine, Jeonbuk National University, Jeonju 54907, Korea; 3 Biomedical Research Institute, Jeonbuk National University Hospital, Jeonju 54907, Korea; 4 Department of Surgery, Jeonbuk National University Medical School, Jeonju 54907, Korea; hirojawa@jbnu.ac.kr

**Keywords:** compressed sensing, gradient and spin echo, breath-hold, magnetic resonance, imaging, cholangiopancreatography, three-dimensional

## Abstract

While magnetic resonance cholangiopancreatography (MRCP) is routinely used, compressed sensing MRCP (CS-MRCP) and gradient and spin-echo MRCP (GRASE-MRCP) with breath-holding (BH) may allow sufficient image quality with shorter acquisition times. This study qualitatively and quantitatively compared BH-CS-MRCP and BH-GRASE-MRCP and evaluated their clinical effectiveness. Data from 59 consecutive patients who underwent both BH-CS-MRCP and BH-GRASE-MRCP were qualitatively analyzed using a five-point Likert-type scale. The signal-to-noise ratio (SNR) of the common bile duct (CBD), contrast-to-noise ratio (CNR) of the CBD and liver, and contrast ratio between periductal tissue and the CBD were measured. Paired t-test, Wilcoxon signed-rank test, and McNemar’s test were used for statistical analysis. No significant differences were found in overall image quality or duct visualization of the CBD, right and left 1st level intrahepatic duct (IHD), cystic duct, and proximal pancreatic duct (PD). BH-CS-MRCP demonstrated higher background suppression and better visualization of right (*p* = 0.004) and left 2nd level IHD (*p* < 0.001), mid PD (*p* = 0.003), and distal PD (*p* = 0.041). Image quality degradation was less with BH-GRASE-MRCP than BH-CS-MRCP (*p* = 0.025). Of 24 patients with communication between a cyst and the PD, 21 (87.5%) and 15 patients (62.5%) demonstrated such communication on BH-CS-MRCP and BH-GRASE-MRCP, respectively. SNR, contrast ratio, and CNR of BH-CS-MRCP were higher than BH-GRASE-MRCP (*p* < 0.001). Both BH-CS-MRCP and BH-GRASE-MRCP are useful imaging methods with sufficient image quality. Each method has advantages, such as better visualization of small ducts with BH-CS-MRCP and greater time saving with BH-GRASE-MRCP. These differences allow diverse choices for visualization of the pancreaticobiliary tree in clinical practice.

## 1. Introduction

Since magnetic resonance cholangiopancreatography (MRCP) was first introduced in the 1990s, it has become an established imaging technique for noninvasive examination of the biliary tree and pancreatic duct (PD), including anatomic variations as well as various pathologies such as biliary stone disease, inflammation, and malignancy [1,2,3,4,5,6]. Further, three-dimensional MRCP (3D-MRCP) may be superior to 2D MRCP due to its isotropic voxel size and nonorthogonal projections [7].

However, since conventional 3D-MRCP uses a respiratory triggered (RT) heavily T2-weighted fast or turbo spin-echo sequence [4,8,9], long acquisition times are hurdles as the 3D-MRCP acquisition time makes up a large amount of the entire examination time [9,10]. Especially in patients with irregular breathing, acquisition times can be extended to 10 min or longer, resulting in RT 3D-MRCP measurements with increased image blurring and motion artifacts, which often results in suboptimal image quality [11]. Thus, alternative methods have been suggested to reduce the 3D image acquisition time, such as the 3D gradient- and spin-echo (GRASE) technique [12], 3D balanced steady-state free-precession (b-SSFP) [13], fast 3D T2-weighted turbo spin-echo (TSE) [14], or fast recovery fast spin-echo (FRFSE) [15] sequences. The clinical potential of GRASE-MRCP has increased in the past few years due to improvements in MR hardware, such as a more homogeneous B0 field and a more powerful gradient system with enhanced fidelity [16]. In addition, the recent development of the compressed sensing (CS) technique with sparsity-based magnetic resonance imaging (MRI) [17,18,19] has provided an alternative option for achieving ultrafast 3D-MRCP, with promising results demonstrated in recent studies. However, most studies evaluated a vendor-specific sampling pattern and reconstruction method and lacked quantitative analysis between different techniques or vendors [20,21]. A recent study comparing BH-CS-MRCP and BH-GRASE-MRCP was also limited in that it used the same MR machine without quantitative analysis [22]. Therefore, the purpose of this study was to compare BH-CS-MRCP and BH-GRASE-MRCP acquired from different MR vendors with an emphasis on both qualitative and quantitative analysis.

## 2. Materials and Methods

### 2.1. Patients

This retrospective study was approved by our hospital’s institutional review board, and written informed consent was waived. From October 2018 to August 2019, 59 consecutive outpatients (21 men and 38 women; mean age, 63 years; age range, 25–81 years) underwent both BH-CS-MRCP and BH-GRASE-MRCP. Indication of MRCP were as follows: biliary or pancreatic ductal dilatation (*n* = 11), branch-duct-type intraductal papillary mucinous neoplasm (BD-IPMN) (*n* = 24), cystic pancreatic lesion other than BD-IPMN (*n* = 8), previous history of cholangitis or pancreatitis (*n* = 2), no abnormality in pancreaticobiliary system (*n* = 10), anatomical variation (*n* = 1), chronic cholecystitis (*n* = 1), adenomyomatosis (*n* = 1), and previous history of hepatic dysfunction (*n* = 1). The reference standard for the diagnosis of pancreaticobiliary pathology was based on subsequent endoscopic retrograde cholangiopancreatography (ERCP), endoscopic ultrasound (EUS), surgery, or follow-up images.

### 2.2. MR Examination

All MR examinations were performed on a 3T MR scanner (Magnetom Skyra, Siemens Healthineers, Erlangen, Germany and Achieva, Philips Healthcare, Best, The Netherlands) using an 18-channel body matrix coil combined with a 32-channel spine matrix coil for Skyra and 32-channel flexible anteroposterior phased-array coil for Achieva. Patients fasted for at least 4 h before the examination. No spasmolytic drug or negative oral contrast was used. Both BH-MRCP were acquired within an interval of 3–10 days (mean, 5 days), and the order of acquisition of BH-CS-MRCP and BH-GRASE-MRCP was randomized.

### 2.3. Compressed Sensing MRCP

Compressed sensing MRCP used a prototypical 3D SPACE sequence with an incoherent undersampling scheme and CS reconstruction technique (CS SPACE, Siemens Healthineers, Erlangen, Germany). Incoherent undersampling was obtained in this prototype sequence with a Poisson-disk pattern in 2 phase-encoding dimensions [19]. Fluctuations in echo train trajectories arising from irregular k-space sampling were modulated by increasing the smoothness of train trajectories [23]. The specific acquisition parameters were as follows: FOV, 384 × 192 mm^2^; TR/TE, 1700/503 milliseconds; FA, 110 degrees; spectrally selective fat saturation to saturate fat signal intensity; NEX, 2; section thickness, 1 mm; resolution (interpolated), 1 × 1 × 1 mm (0.5 × 0.5 × 1 mm); and a number of coronal sections, 72. We set the scan range to cover the whole biliary system by setting the center of sequence at the bifurcation of the right and left IHD. The acquisition time was 16 s. The CS reconstruction technique used 14 iterations, an acceleration factor of 17 (5.7% k-space data sampling), and a regularization parameter of 0.003. The inline-image reconstruction took about 4–5 min for each data set.

### 2.4. Gradient and Spin-Echo MRCP

Images were obtained using a single breath-hold 3D MRCP with a GRASE sequence [12]. To minimize the specific absorption rate (SAR) and reduce TR, a refocusing angle of 100° was introduced after the primary 180° pulse. Combining the parallel acceleration and echo-planar imaging (EPI) factors reduced distortion. Detailed acquisition parameters were as follows: FOV, 330 × 330 mm^2^; TR/TE, 268/87.1 milliseconds; FA, 90 degrees; NEX, 1; section thickness, 2 mm; resolution (interpolated), 1.37 × 1.37 × 2 mm (0.64 × 0.64 × 1 mm); parallel acceleration factor, 2; turbo factor, 15; EPI factor, 7; and number of coronal sections, 90. Same as BH-CS-MRCP, we set the scan range to cover the whole biliary system by setting the center of sequence at the bifurcation of the right and left IHD. The acquisition time was 12 s.

### 2.5. Image Analysis

#### 2.5.1. Qualitative Image Analysis

Three radiologists independently examined BH-CS-MRCP and BH-GRASE-MRCP images within a 4-week interval to reduce recall bias (two rounds of qualitative analysis). Each image was anonymized and distributed to the reviewers randomly, and the readers were blinded to details of the acquisition process. The radiologists were allowed to modify the width and window level during the analysis. However, no data from other MR sequences were seen by the radiologists.

A 5-point Likert-type scale based on a previous study was used for the qualitative analysis. The scale included the following four parts: background suppression, artifacts, duct visualization, and overall image quality, as shown in Table 1. For evaluating duct visualization, the whole pancreaticobiliary system was segmented into 9 sections as follows: the CBD; cystic duct insertion; bilateral first and second IHD (intrahepatic duct); and PD in the proximal, middle, and distal segments. For each section, ductal visualization was evaluated using a 5-point Likert-type scale (Table 1). Communication between a cyst and the PD was evaluated for 24 patients who had BD-IPMN through analyzing the 3D images in addition to the BH-CS-MRCP and BH-GRASE-MRCP source images.

#### 2.5.2. Quantitative Image Analysis

**Figure 1:**
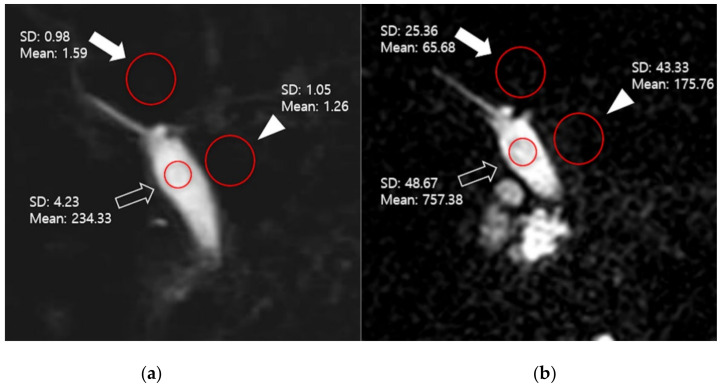
A representative slice of breath-holding (BH)-CS-compressed sensing MRCP (MRCP) (**a**) and BH- gradient and spin-echo MRCP (GRASE-MRCP) (**b**) used for quantitative analysis (upper CBD: hollow arrow, periductal tissue: arrowhead, liver: solid arrow). The mean value of regions of interest (ROI) (red circles) represents signal intensity (SI), and standard deviation (SD) was used as image noise. Signal-to-noise ratio, contrast ratio, and contrast-to-noise ratio were 45.26, 0.98, 62.4 on BH-CS-MRCP(**a**), and 23.15, 0.69, 23.61 on BH-GRASE-MRCP (**b**), respectively.

A research associate (2 years of experience) provided the quantitative imaging analysis of the source images (two times over a 4-week interval). The mean values were used for the analysis. Using an approach like described in other previous studies, three representative slices (upper, middle, and lower CBD) depicting the center of the CBD for each patient were determined, and regions of interest (ROIs) were used to measure the signal intensity (SI) of the CBD and periductal tissues. ROIs for the SI of bile of at least 5 mm^2^ were used in homogeneous and artifact-free regions of the CBD in the middle third of its course. ROIs for the SI of periductal tissue and liver of at least 20 mm^2^ were placed in homogeneous and artifact-free regions adjacent to the ROI of the CBD [24,25]. Since the background noise was low, image noise was defined as the standard deviation (SD) of the CBD, periductal tissue, and the liver from the same ROIs as those used for SI measurements (Figure 1). The following formulae were used to evaluate the signal-to-noise ratio (SNR) of the CBD and the contrast ratio between the CBD and periductal tissues on 3D MRCP:


SNR = SI_CBD_/SD_CBD_

Contrast ratio = (SI_CBD_ − SI_periductal tissue_)/(SI_CBD_ + SI_periductal tissue_)

In accordance with previous reports [25], the contrast-to-noise ratio (CNR) between the CBD and the liver was calculated using the following formula:


CNR = (SI_CBD_ − SI_liver_)/{[(SD_CBD_)^2^ + (SD_liver_)^2^]/2}^1/2^

### 2.6. Statistical Analyses

Numerical values were presented as mean ± standard deviation. After the normality test, either paired t-test or Wilcoxon signed-rank test was used as appropriate to analyze qualitative differences between BH-CS-MRCP and BH-GRASE-MRCP scores. SNR, contrast ratio and CNR were analyzed with a paired t-test. McNemar’s test was used to analyze the communication between a cyst and the PD. Interobserver agreement was analyzed for each qualitative item using intraclass correlation coefficients with a two-way model. Intraclass correlation coefficient values were classified as poor (<0.40), fair to good (0.40–0.75), or excellent (>0.75) [26]. Statistical analyses were performed using MedCalc version 18.6 (MedCalc Software, Ostend, Belgium). A *p*-value of <0.05 was considered statistically significant.

## 3. Results

### 3.1. Qualitative Image Analysis

**Figure 2:**
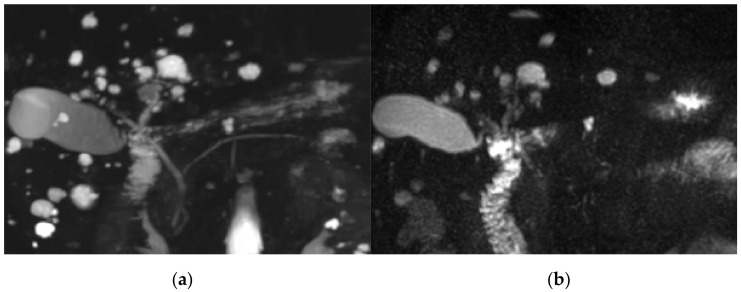
A 64-year-old female with multiple hepatic cysts. Image degradation by artifacts (4.7 vs. 4, BH-CS-MRCP vs. BH-GRASE-MRCP, respectively), background suppression (5 vs. 3.7), overall image quality (5 vs. 2.7,), visualization of common bile duct (CBD) (4.7 vs. 3), right 1st intrahepatic duct (IHD) (4.7 vs. 4), left 1st IHD (4.7 vs. 4), right 2nd IHD (4 vs. 2), cystic duct insertion (4.3 vs. 3.7), proximal *p*-duct (4.3 vs. 1.3), middle *p*-duct (5 vs. 1.7), and distal *p*-duct (4.7 vs. 1) were better with BH-CS-MCRP (**a**) than those with BH-GRASE-MRCP (**b**).

**Figure 3:**
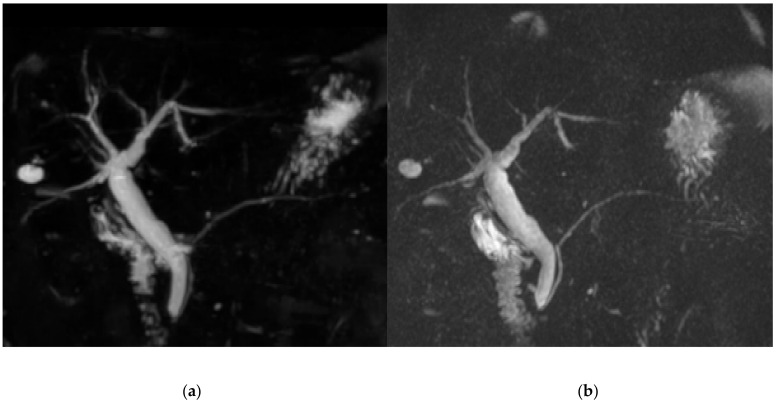
An 80-year-old female who had a history of pancreatitis. Image degradation by artifacts (5 vs. 5, BH-CS-MRCP vs. BH-GRASE-MRCP, respectively) and overall image quality (5 vs. 5) were comparable between BH-CS-MRCP (**a**) BH-GRASE-MRCP (**b**). Background suppression (4.7 vs. 4.3) was better with BH-CS-MRCP (**a**) than those with BH-GRASE-MRCP (**b**).

**Figure 4:**
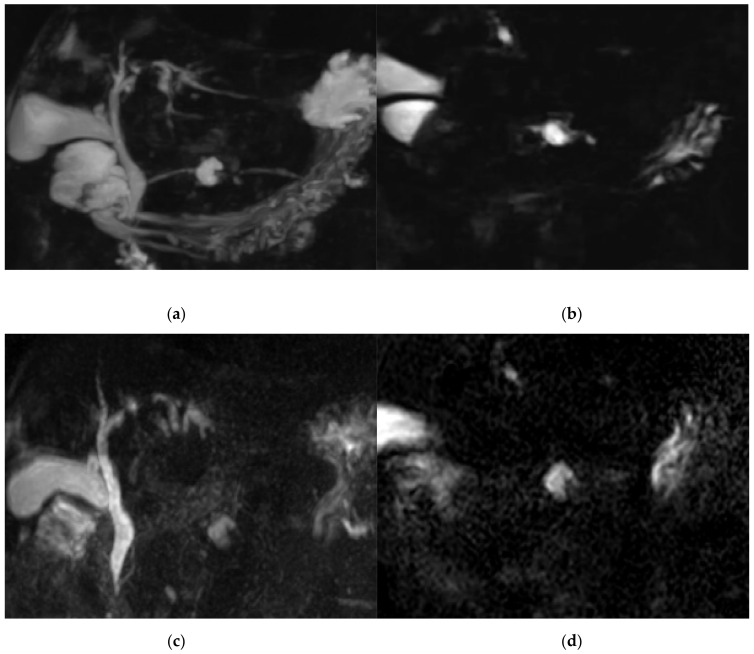
A 79-year-old female with BD-IPMN in pancreatic body. Communication between a cyst and proximal pancreatic duct (PD) was shown with BH-CS-MRCP in both the 3D (**a**) and source image (**b**), but not shown with BH-GRASE-MRCP in both the 3D (**c**) and source (**d**) image. Visualization of proximal PD (5 vs. 5, BH-CS-MRCP vs. BH-GRASE-MRCP, respectively), mid PD (4 vs. 1.7), and distal PD (4 vs. 1.7) were better with BH-CS-MRCP (**a**) than those with BH-GRASE-MRCP (**c**).

The score for image quality degradation by artifacts was significantly higher with BH-GRASE-MRCP than BH-CS-MRCP (4.86 ± 0.26 vs. 4.71 ± 0.42, *p* = 0.025). Background suppression, visualization of right and left 2nd level IHD, mid PD, and distal PD were significantly higher with BH-CS-MRCP than BH-GRASE-MRCP (*p* < 0.05, Table 2, Figure 2). However, overall image quality (*p* = 0.797), duct visualization of the CBD (*p* = 0.242), right (*p* = 0.589) and left 1st level IHD (*p* = 0.238), cystic duct (*p* = 0.089), and proximal PD (*p* = 0.643) did not show a significant difference between the imaging modalities (Figure 3). There were 24 patients with BD-IPMN who were considered to have communication between a cyst and the PD, while 21 patients (87.5%) on BH-CS-MRCP and 15 patients (62.5%) on BH-GRASE-MRCP demonstrated the presence of such communication (*p* = 0.07, Table 2, Figure 4).

The interobserver agreement was fair to excellent for all items on BH-CS-MRCP (ICC = 0.47–0.94) and BH-GRASE-MRCP (ICC = 0.52–0.95) except background suppression, which showed poor interobserver agreement on both BH-CS-MRCP (ICC = 0.35) and BH-GRASE-MRCP (ICC = 0.31, Table 3).

### 3.2. Quantitative Image Analysis

The SNR of the CBD with BH-CS-MRCP was 56.5% higher than that with BH-GRASE-MRCP (*p* < 0.001). The contrast ratio between the CBD and periductal tissue with BH-CS-MRCP was 31.9% higher than that with BH-GRASE-MRCP, and the CNR between the CBD and the liver with BH-CS-MRCP was 104.8% higher than that with BH-GRASE-MRCP (*p* < 0.001, Table 4).

## 4. Discussion

In this study, we compared BH-CS-MRCP with BH-GRASE-MRCP through qualitative and quantitative assessment. To our knowledge, this is the first study to compare both BH-MRCP techniques using equipment from different MR vendors. No significant differences in qualitative image quality were found between the two techniques, but background suppression was better with BH-CS-MRCP. On the other hand, the score for image degradation by artifacts was higher with BH-GRASE-MRCP, although both scores were high (4.86 ± 0.26 for BH-GRASE-MRCP and 4.71 ± 0.42 for BH-CS-MRCP, respectively). Despite similar overall image quality, BH-CS-MRCP performed significantly better than BH-GRASE-MRCP for the visualization of both secondary IHDs and mid to distal PDs. We also found a trend for better visualization of communication between a cyst and the PD with BH-CS-MRCP.

Although BH-CS-MRCP has shown great potential as an alternative method of RT MRCP, earlier studies using BH-CS-MRCP have reported several issues, such as inferior background signal suppression and lesser visibility of small ductal structures similar to GRASE-MRCP. However, we used an optimized BH-CS-MRCP with a smaller FOV and reduced acceleration factor, which was proven to be equally good or even better than conventional MRCP [27,28]. Some caution is needed when interpreting our study results because a recent study comparing BH-CS-MRCP and BH-GRASE-MRCP using the same MR machine with a BH time of 16–20 s showed no significant difference in image quality except for background suppression [22]. The EPI factor or acquisition time needs to be increased to have a better spatial resolution for GRASE [29]. If our BH-GRASE-MRCP used a longer BH time with fewer acceleration factors, the results might have been similar. Further studies using variable acquisition parameters for BH-GRASE-MRCP and BH-CS-MRCP are warranted.

Other than a longer acquisition time for BH-GRASE-MRCP, another difference between He et al., and our study is that the previous study used negative oral contrast media to suppress T2 signals of the gastrointestinal tract, while we did not use any oral contrast media [22]. Chien et al., assume that negative oral contrast media could cause an increased background signal in BH-GRASE-MRCP [20]. Although direct comparison of qualitative scores between the two different studies is difficult, background suppression for BH-GRASE-MRCP was better in our study compared to He et al., (4.02 vs. 3.885), while background suppression for BH-CS-MRCP was quite similar (4.46 vs. 4.628). As GRASE is inherently a combination of gradient echo and FSE [30], susceptibility effects in proximity to air-tissue interfaces arising from the gradient-echo [31] could lead to unwanted signal loss and thus may hide the small duct, such as 2nd level IHD or PD. Moreover, GRASE has been reported to show blurring related to the point-spread-function arising from T2 and T2* decay [32], which, with matrix size settings during a limited imaging time, impairs spatial resolution and could be inadequate for showing small ducts [20]. Since our BH-GRASE-MRCP was acquired within 12 s, which is shorter than previous studies (16–20 s) [22,29], we cautiously assume that the aforementioned factors may have resulted in inferior visualization of the right and left 2nd IHD as well as mid to distal PD compared to BH-CS-MRCP in the current study.

BH-CS-MRCP necessitates a uniquely determined pulse sequence using high-performance hardware and software, which can limit its applicability to only high-end MR machines that have been recently developed. Complex reconstruction of the CS technique also necessitates a higher computational power, and Hence, the reconstruction speed may present a limitation for the workflow. In contrast, BH-GRASE-MRCP does not require additional time for image reconstruction, Hence, even though image acquisition time is comparable as both are acquired in one BH, the overall time required for the final image acquisition is much shorter for BH-GRASE-MRCP. In addition, we set the BH time of BH-GRASE-MRCP as 12 s, which was reduced by more than 20% compared with recent studies [22,29]. Since this time-saving method retained good image quality, it would be useful for patients who cannot hold their breath adequately, such as emergency patients with acute abdominal pain or in patients with a recent history of invasive pancreaticobiliary procedures (e.g., ERCP, percutaneous transhepatic biliary drainage). However, there is one major limitation of BH-GRASE-MRCP; it is currently available by only one specific vendor. Hence, the applicability of BH-GRASE-MRCP for various vendors still needs to be proven [16]. Considering the short acquisition time and excellent image quality for visualizing the pancreaticobiliary system, both BH-CS and BH-GRASE-MRCP could be considered as first-line imaging modalities if both sequences are available. Since each technique has different characteristics, however, the choice could be flexible according to the attending radiologist’s preferences or specific clinical conditions of patients (e.g., BH-CS-MRCP should be used first in patients suspected with PD abnormalities, and BH-GRASE-MRCP should be used first in acutely ill patients with decreased BH capability).

The SNR, contrast ratio, and CNR measured by BH-CS-MRCP were significantly higher than those measured by BH-GRASE-MRCP in this study. While the SI of the CBD, liver, and periductal tissue were reduced on BH-CS-MRCP in comparison to those on BH-GRASE-MRCP, the notable decrease in image noise led to a better ratio in quantitative analysis. An earlier study demonstrated that visualizing the main pancreatic duct was limited due to a larger background signal. As signals from the background and surrounding organs are higher with a short TE [25,33], this may explain the higher SI of periductal tissue and lower SNR of BH-GRASE-MRCP, which uses a much shorter TE than BH-CS-MRCP. Even though there was a significant difference in the quantitative analysis results between BH-CS-MRCP and BH-GRASE-MRCP, there was no significant difference in overall image quality. This may because MRCP images are associated with high T2 contrast for fluid signals in pancreaticobiliary structures; hence, potentially changing the image texture and artifact patterns likely would not significantly alter the image quality of MRCP [34,35,36]. In addition, since the basic principle as well as the acquisition parameters of BH-CS-MRCP and BH-GRASE-MRCP are different, cautious interpretation of the results is warranted.

Our study has several limitations. First, this was a single-center retrospective study with a relatively small number of patients, and thus the results should be interpreted cautiously. Second, diagnostic performance could not be analyzed due to the small number of acceptable cases. As a result, we concentrated on qualitative and quantitative image analysis. Third, since calculating parameters, such as SNR and CNR, in parallel imaging or compressed sensing is technically difficult and often not clinically feasible, the findings of our study should be carefully interpreted [37]. Fourth, we lacked quantitative analysis, such as measuring the diameter of ductal structures or volume of pancreaticobiliary structures. A recent study suggested using dedicated software to improve repeatability and reproducibility of MRCP since MRCP is limited by its qualitative nature of interpretation resulting in high rates of inter- and intra-rater variation [38,39]. In addition, as denoising techniques have been developing, various quantitative analyses would enhance MRCP as a more objective imaging method with higher SNR. Future studies comparing BH-CS-MRCP and BH-GRASE-MRCP regarding these issues is warranted.

## 5. Conclusions

In conclusion, both BH-CS-MRCP and BH-GRASE-MRCP are useful image acquisition methods that can be acquired with a single breath-hold while maintaining adequate image quality. Each method has characteristic advantages, such as better background suppression and visualization of small ducts by BH-CS-MRCP and less artifact and greater time saving with BH-GRASE-MRCP. These detailed differences allow for a diverse choice in clinical practice for the visualization of the pancreaticobiliary tree.

## Figures and Tables

**Table 1:** Details of scoring scale in the assessment for various parameters.

Parameter	Score	Scoring System
Image quality degradation by artifacts	1	Nondiagnostic image due to severe artifacts
	2	Major artifacts causing significant problems in diagnosis
	3	Moderate artifacts with some uncertainty in diagnosis
	4	Minor artifacts without problems in diagnosis
	5	Excellent image quality without any detectable artifacts
Background suppression	1	Significant background signal that rendered image interpretation impossible
	2	Remarkable background signal that rendered image interpretation difficult
	3	Noticeable background signal that is distracting in image interpretation
	4	Minimal background signal without problems in observation of pancreaticobiliary tree
	5	Excellent background suppression
Overall image quality	1	Nondiagnostic image
	2	Below average image quality
	3	Average image quality
	4	Good image quality
	5	Excellent image quality
Ductal visualization	1	Ductal structure not visible
	2	Ductal structure vaguely identified
	3	Ductal structure partially visible
	4	Most of the ductal structure visible, with some blurring
	5	Entire ductal structure visible with excellent details

**Table 2:** Comparison of image quality between BH-CS-MRCP and BH-GRASE-MRCP.

	BH-CS-MRCP	BH-GRASE-MRCP	*p*-Value *
Artifact	4.71 ± 0.42 (3.0–5.0)	4.86 ± 0.26 (4.0–5.0)	0.025
Background suppression	4.46 ± 0.39 (3.7–5.0)	4.02 ± 0.28 (3.0–4.7)	<0.001
Overall image quality	4.23 ± 0.61 (2.3–5.0)	4.21 ± 0.66 (2.3–5.0)	0.797
Duct visualization			
CBD	4.78 ± 0.36 (3.7–5.0)	4.67 ± 0.54 (3.0–5.0)	0.242
Right 1st IHD	4.62 ± 0.43 (3.3–5.0)	4.64 ± 0.62 (3.3–5.0)	0.589
Left 1st IHD	4.66 ± 0.39 (3.0–5.0)	3.42 ± 0.77 (3.0–5.0)	0.238
Right 2nd IHD	3.85 ± 0.70 (2.0–5.0)	3.48 ± 0.97 (2.0–5.0)	0.004
Left 2nd IHD	3.81 ± 0.64 (2.3–5.0)	3.42 ± 0.77 (2.3–5.0)	<0.001
Cystic duct	4.02 ± 0.73 (2.3–5.0)	4.22 ± 0.65 (2.3–5.0)	0.089
Proximal PD	4.05 ± 1.07 (1.0–5.0)	3.89 ± 1.32 (1.0–5.0)	0.643
Mid PD	3.99 ± 1.17 (1.0–5.0)	3.55 ± 1.41 (1.0–5.0)	0.003
Distal PD	3.71 ± 12.5 (1.0–5.0)	3.38 ± 1.42 (1.0–5.0)	0.041
Communication between PD and cyst	21/24 ^†^ (87.5%)	15/24 ^†^ (62.5%)	0.070

* *p*-value < 0.05 indicate statistically significant differences. Values are mean ± standard deviation (range) or numeric ^†^. MRCP, magnetic resonance cholangiopancreatography; BH, breath-hold; CS, compressed sensing; GRASE, gradient and spin echo; CBD, common bile duct; IHD, intrahepatic bile duct; PD, pancreatic duct.

**Table 3:** Intraclass correlation coefficients of qualitative analysis on BH-CS-MRCP and BH-GRASE-MRCP.

	BH-CS-MRCP	BH-GRASE-MRCP
Artifact	0.74 (0.60, 0.84)	0.52 (0.26, 0.70)
Background suppression	0.35 (0.00, 0.59)	0.31 (−0.07, 0.57)
Overall image quality	0.72 (0.57, 0.82)	0.79 (0.67, 0.87)
Duct visualization		
CBD	0.71 (0.55, 0.82)	0.88 (0.82, 0.93)
Right 1st IHD	0.64 (0.44, 0.77)	0.86 (0.78, 0.91)
Left 1st IHD	0.47 (0.18, 0.67)	0.70 (0.54, 0.81)
Right 2nd IHD	0.72 (0.57, 0.83)	0.84 (0.75, 0.90)
Left 2nd IHD	0.67 (0.49, 0.79)	0.71 (0.55, 0.82)
Cystic duct	0.81 (0.69, 0.88)	0.73 (0.56, 0.83)
Proximal PD	0.92 (0.88, 0.95)	0.94 (0.91, 0.96)
Mid PD	0.93 (0.89, 0.96)	0.95 (0.93, 0.97)
Distal PD	0.94 (0.90, 0.96)	0.95 (0.92, 0.97)

ICC with its 95% confidence interval. MRCP, magnetic resonance cholangiopancreatography; BH, breath-hold; CS, compressed sensing; GRASE, gradient and spin echo; CBD, common bile duct; IHD, intrahepatic bile duct; PD, pancreatic duct.

**Table 4:** Results of quantitative analysis.

	BH-CS-MCRP	BH-GRASE-MRCP	*p*-Value *
T2 signal intensity			
CBD	251.49 ± 71.99	821.95 ± 202.03	<0.001
Periductal tissue	4.04 ± 2.22	124.69 ± 43.48	<0.001
Liver	1.69 ± 2.51	71.83 ± 19.40	<0.001
Noise			
CBD	11.01 ± 4.12	75.66 ± 187.53	0.01
Periductal tissue	2.93 ± 1.45	39.66 ± 9.17	<0.001
Liver	0.73 ± 1.20	28.52 ± 8.29	<0.001
Signal-to-noise ratio	26.86 ± 14.58	17.16 ± 6.38	<0.001
Contrast ratio	96.70 ± 0.02	73.30 ± 0.07	<0.001
Contrast-to-noise ratio	37.48 ± 20.27	18.30 ± 6.21	<0.001

* *p*-value < 0.05 indicate statistically significant differences. Values are mean ± standard deviation. MRCP, magnetic resonance cholangiopancreatography; BH, breath-hold; CS, compressed sensing; GRASE, gradient and spin echo; CBD, common bile duct; IHD, intrahepatic bile duct; PD, pancreatic duct.
